# A non-mutated *TROP2* fingerprint in cancer genetics

**DOI:** 10.3389/fonc.2023.1151090

**Published:** 2023-06-29

**Authors:** Emanuela Guerra, Roberta Di Pietro, Gianmarco Stati, Saverio Alberti

**Affiliations:** ^1^ Laboratory of Cancer Pathology, Center for Advanced Studies and Technology (CAST), “G. d’Annunzio” University of Chieti-Pescara, Chieti, Italy; ^2^ Department of Medical, Oral and Biotechnological Sciences, “G. d’Annunzio” University of Chieti-Pescara, Chieti, Italy; ^3^ Department of Medicine and Aging Sciences, Section of Biomorphology, G. d’Annunzio University of Chieti-Pescara, Chieti, Italy; ^4^ Sbarro Institute for Cancer Research and Molecular Medicine, Center for Biotechnology, Department of Biology, College of Science and Technology, Temple University, Philadelphia, PA, United States; ^5^ Unit of Medical Genetics, Department of Biomedical Sciences - Biomedical Sciences (BIOMORF), University of Messina, Messina, Italy

**Keywords:** gelatinous drop-like corneal dystrophy (GDLD), pancreatic cancer (PC), Trop-2, genomic mutations, gene expression profiles, tumor progression

## Abstract

The advent of high throughput DNA sequencing is providing massive amounts of tumor-associated mutation data. Implicit in these analyses is the assumption that, by acquiring a series of hallmark changes, normal cells evolve along a neoplastic path. However, the lack of correlation between cancer risk and global exposure to mutagenic factors provides arguments against this model. This suggested that additional, non-mutagenic factors are at work in cancer development. A candidate determinant is *TROP2*, that stands out for its expression in the majority of solid tumors in human, for its impact on the prognosis of most solid cancers and for its role as driver of cancer growth and metastatic diffusion, through overexpression as a wild-type form. The Trop-2 signaling network encompasses CREB1, Jun, NF-κB, Rb, STAT1 and STAT3, through induction of cyclin D1 and MAPK/ERK. Notably, Trop-2-driven pathways vastly overlap with those activated by most functionally relevant/most frequently mutated *RAS* and *TP53*, and are co-expressed in a large fraction of individual tumor cases, suggesting functional overlap. Mutated Ras was shown to synergize with the *TROP2-CYCLIND1* mRNA chimera in transforming primary cells into tumorigenic ones. Genomic loss of *TROP2* was found to promote carcinogenesis in squamous cell carcinomas through modulation of Src and mutated Ras pathways. DNA methylation and *TP53* status were shown to cause genome instability and *TROP* gene amplification, together with Trop-2 protein overexpression. These findings suggest that mutagenic and the *TROP2* non-mutagenic pathways deeply intertwine in driving transformed cell growth and malignant progression of solid cancers.

## 1 Introduction

The advent of high throughput DNA sequencing is providing massive amounts of data on tumor-associated mutations (1) and on the landscape of genetic alterations in cancer (2). Oncogene/tumor suppressor gene mutation models have been adopted as a conceptual framework for multi-stage tumor progression. Underlying these analyses there is the assumption that normal cells progressively evolve along the tumor progression path (3). In contrast with this model, no correlation can be found between cancer risk and body size (i.e. number of cell replications) or longevity (i.e. duration of exposure to mutagenic factors), which is known as the Peto’s paradox (4).

This suggested that non-mutagenic mechanisms (5) cooperate with mutagenic oncogenic events (6) to drive tumor progression. Trop-2 is a key candidate for such mechanisms (7). Upregulation of Trop-2 has been associated to poor prognosis of lung (8), breast (9), pancreas (10, 11), stomach (12), head and neck (13), ovary (14) and colon-rectum cancers (11), suggesting a pivotal role of this molecule in tumor progression (15) and metastatic diffusion (16). Notably, the Trop-2 non-mutagenic signature of cancer-driving signaling networks (17) appears to vastly overlap with signatures of mutated oncogenes (18). Experimental evidence for this has been obtained in *mTROP2* knockout mouse models (19) and in human tumors, whereby interaction between Cyclin D1, *TROP2* and mutated *RAS* leads to the transformation of primary, naïve cells into tumorigenic ones (20). DNA methylation and *TP53* status were additionally shown to cause genome instability, *TROP* gene amplification and Trop protein overexpression (21). Thus, mutagenic and *TROP2* non-mutagenic pathways may deeply intertwine in driving cancer cell growth, and play a convergent role in tumor progression in solid cancers.

## 2 Impact of Trop-2 in cancer and genetic diseases

### 2.1 Trop-2 in cancer

*TROP2* is a candidate non-mutated cancer driver, that stands out for its expression in the majority of solid tumors in human (7, 22, 23). Trop-2 (AC: P09758) is a type-I transmembrane protein, encoded by the tumor-associated calcium signal transducer 2 (*TROP2/TACSTD2/M1S1/GA733-1*) gene (7, 24, 25), a retrotransposon of the *TROP1/TACSTD1/EPCAM* gene (24, 26). The extracellular domain of Trop-2 (residues 27–274 in human) encompasses a cysteine-rich N-terminal region, which hosts a GA733 type 1 motif (residues 27-69) (27), and a thyroglobulin type-1 domain (residues 70-148), followed by a C-terminal domain devoid of cysteines (residues 149-274) (7). The 26-amino acid intracellular domain contains a HIKE motif (28), and two PKC phosphorylation sites, at Ser303 and Ser322 (29).

**Figure 1:**
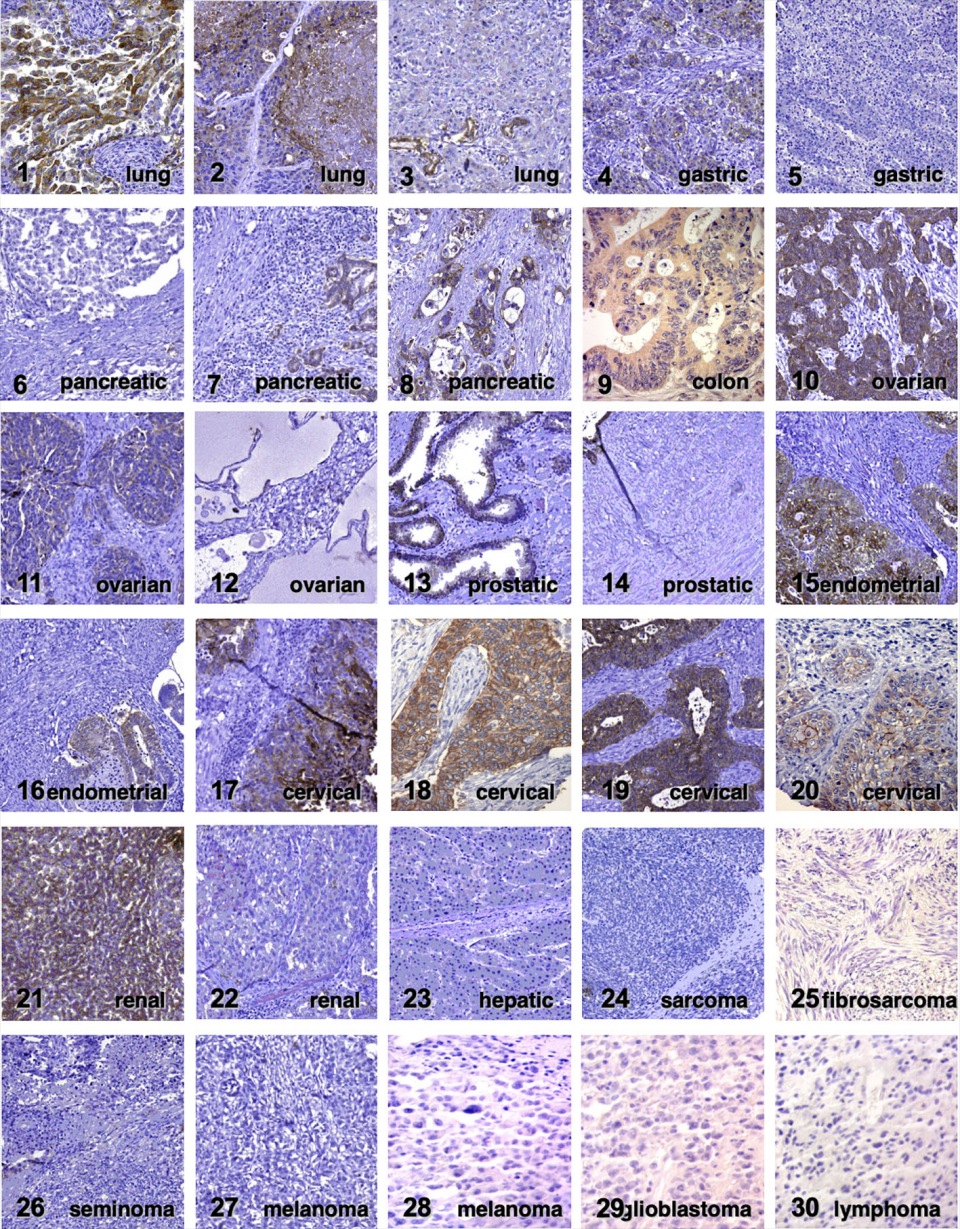
Trop-2 expression in cancer. Immunohistochemical analysis of Trop-2 protein expression in representative tumor cases (23). Tumor histotypes are indicated. Four expression subgroups (absent, weak, moderate and intense) were defined according to Spizzo et al. (31). Negative tumors are cases 5, 6, 12, 14, 22-30. Weak expression was detected in cases 7, 11, 20. Moderate expression was found in cases 3, 4, 8, 13, 16, 21. Intense expression was observed in cases 1, 2, 9, 10, 15, 17-19.

**Figure 2:**
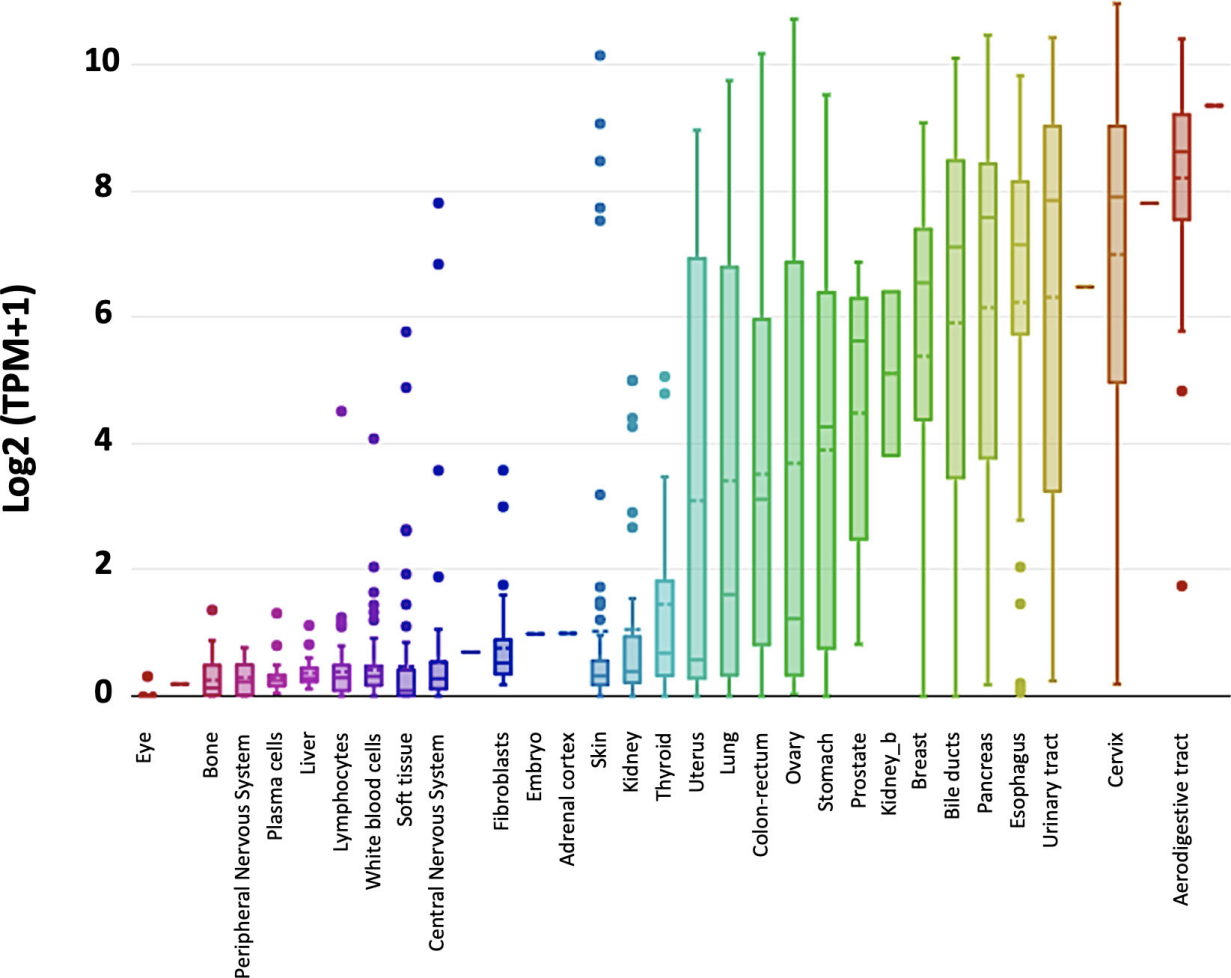
*TROP2* gene expression in normal tissues. *TROP2* mRNA expression levels were obtained from the DepMap portal (https://depmap.org/portal/) (Expression public 20Q2) and expressed as Log2 (TPM+1). Whisker plots show value distributions of *TROP2* mRNA levels across different normal tissues. Boxes corresponding to individual tissues are color coded according to their median intensity (horizontal bars within each box). Dots indicate outlier measurements.

Trop-2 induces tumor (23) and cancer stem cell growth (30). Our findings showed that Trop-2 expression is upregulated in tumors, regardless of baseline expression in normal tissues (23). Representative portraits of Trop-2 protein in 30 neoplasia types are shown in **Figure 1**. *TROP2* mRNA expression levels in normal tissues are shown in **Figure 2**. These findings first suggested that Trop-2 expression in cancer cells provided a selective advantage. Consistent with this, upregulation of wtTrop-2 was shown to stimulate tumor growth in proportion to expression levels *in vivo* (23). This correspondingly raised interest in Trop-2 as a target for immunotherapy in solid cancer (32, 33), and in the development of next-generation anti-Trop-2 antibodies (34).

Using antibody targeting and N-terminal Edman degradation, we showed that Trop-2 undergoes cleavage in the first loop of the thyroglobulin domain in the extracellular region, between residues R87 and T88. ADAM10 was shown to be effector protease at this site (16). Molecular modeling indicated that this cleavage induces a profound rearrangement of the Trop-2 structure, which suggested impact on its biological function. No Trop-2 cleavage was detected in normal human tissues, whereas most tumors, including skin, ovary, colon and breast cancers, showed Trop-2 proteolysis. Proteolysis of Trop-2 at R87-T88 was shown to trigger cancer cell growth and metastatic spreading (11).

### 2.2 Trop-2 alterations cause a rare genetic disease

Given the role of Trop-2 in cancer, a role in cancer inheritance was explored. Genomic loss of the murine *TROP2* (*mTrop2*) gene was shown to promote carcinogenesis in squamous cell carcinomas through modulation of Arf, Src and mutated Ras pathways in *mTrop2* mouse knockouts (19).

Genomic alterations of the human *TROP2* were shown to cause a rare genetic disease, whereby *TACSTD2* mutations induce an amyloid corneal dystrophy (Gelatinous Drop-like Corneal Dystrophy, GDLD) (35), while other organs that host Trop-2-expressing epithelia remain devoid of amyloid deposits. A common GDLD mutation is the substitution of the codon for glutamine at position 118 with a stop codon, in Japan (36) and worldwide (37). However, a broad spectrum of mutations has been subsequently detected (38), and novel, more rare mutations are still being added to this list (39). No clear association of GDLD mutations to altered cancer incidence has been reported (23).

### 2.3 Epigenetic *TROP2* signatures in cancer

The expression of the *TROP2* gene was found to be under epigenetic control in choriocarcinomas, i.e. trophoblast-derived malignancies (40, 41). This was shown by associating the expression of *TROP1, TROP2* and control genes to their native genomic configuration and DNA methylation status in choriocarcinoma cells. In other words, intact gene coding capacity was shown by transfection and expression of the encoded protein, as modulated by their respective DNA methylation status (40). Epigenetic control was shown for HLA class I genes, for the T-cell differentiation antigens CD5 and CD8, for *TROP1* and *TROP2*. Treating choriocarcinoma cells with the lowest expression ability with 5-azacytidine led to DNA demethylation, and to differential reexpression of cell surface antigen genes. Further cultivation of choriocarcinoma cells in the absence of 5-azacytidine resulted in renewed methylation of their DNA and reversion to baseline low expression capacity (40).

Cases with epigenetic reduction of the expression of the *TROP2* gene were identified in prostate cancer (42). *TACSTD2* was unmethylated in prostatic intraepithelial neoplasia and hypermethylated/down-regulated in 17% of prostate cancers (42). Low *TROP2* expression was observed in lung adenocarcinomas, as compared with normal lung tissues. Bisulphite DNA sequencing and methylation-specific polymerase chain reaction showed that loss of expression was due to hypermethylation of the *TROP2* promoter region. Consistent with this, DNA demethylation with 5-Aza-deoxycytidine led to activation of *TROP2* expression (43). *TROP2* was found highly expressed in normal bile duct epithelia, but down-regulated in cholangiocarcinoma cells. Sixty percent of cholangiocarcinomas revealed *TROP2* promoter hypermethylation and *TROP2* knockdown significantly enhanced the proliferation and migration of cholangiocarcinoma cell lines (44).

### 2.4 Post-transcriptional and post-translational modulation of *TROP2* expression in cancer

The expression of *TROP2* in cancer was shown to be modulated by both post-transcriptional and post-translational mechanisms. A bi-cistronic *CYCLIN D1-TROP2* mRNA chimera was isolated from human ovarian and mammary cancer cells (20). The *CYCLIN D1-TROP2* mRNA was shown to transform naïve, primary cells and to induce aggressive tumor growth. Silencing of the chimeric mRNA inhibited breast cancer growth. The *CYCLIN D1-TROP2* mRNA was found expressed by a large fraction of human gastro-intestinal, ovarian and endometrial tumors. The chimeric mRNA was shown to be of post-transcriptional origin, and independently translated the Cyclin D1 and Trop-2 proteins. Truncation of the 3’ UTR of the *CYCLIN D1* mRNA led to higher mRNA stability, for inappropriate expression during the cell cycle. There was a quantitative correlation between the chimeric mRNA and the transcriptional levels of *CYCLIN D1* and *TROP2*. Hence, an oncogenic determinant appears linked to levels of expression of the parental moieties, in the absence of both epigenetic changes and of mutagenic alterations. This mechanism of cell transformation appears widespread in human cancers (20).

A distinct post-transcriptional *TROP2* modulation mechanism was shown to involve the Trop-2/miR-125b axis in the progression from normal urothelium to non-invasive and invasive urothelial cancer (45). miR-125b inhibits Trop-2 expression. Experimental findings showed that a progressive increase in Trop-2 protein levels along urothelial cancer progression is induced by miR-125b downregulation, and this correlates with the severity of the disease (45).

Post-transcriptional modulation of Trop-2 expression was also found to be dependent on environmental factors. In SW480 colon cancer cells (–),-epigallocatechin-3-gallate (EGCG) affected the post-transcriptional processing of the *TROP2* mRNA, which was quickly and specifically degraded in the presence of EGCG. Furthermore, EGCG was found to suppress Trop-2 expression at a post-translational level in HCT-116 cells, by affecting the stability of the Trop-2 protein (46).

### 2.5 The intertwining of mutagenic and Trop-2 non-mutagenic signatures in cancer

DNA methylation was shown to mediate *TROP* gene copy number variation, with corresponding additional alteration of protein expression levels (40, 41), suggesting a corresponding stronger drive for cancer cell proliferation (23). This was shown by selection for high protein expressors at a clonal level, which was found to lead to progressively higher expression of *CD5, CD8α, TROP2* through gene amplification (41). A lack of *TROP1* gene amplification was shown to depend on a unique pattern of gene methylation. This regulatory mode was lost upon treatment with the DNA demethylating agent 5-azacytidine (41) and demethylated *TROP1* genes were found to amplify efficiently and progressively. Thus, DNA methylation not only regulates the expression of the *TROP* genes (40), but also is a determinant of *TROP* gene amplification in tumor cells (41). Mutations of *TP53* were subsequently shown to induce loss of DNA methylation in the *TROP1* gene, which then led to gene amplification (21). This was reverted by transduction of a *wtTP53* gene or by inducing methylation of the genomic DNA with the Sss I DNA methylase (21), demonstrating a mechanistic link between mutations of the *TP53* tumor suppressor gene, genomic instability/gene copy number variation (47) and overexpression of the *TROP* genes.

The expression of the *TROP2* gene was shown to depend on a large network of transcription factors, that includes p63/p53L, ERG, GRHL1/Get-1, HNF1A/TCF-1, HNF4A, SPI1/PU.1, WT1, GLIS2, AIRE, FOXM1 and FOXP3 (17). *TROP2* upregulation was found to then drive the expression and activation of CREB1, Jun, NF-κB, Rb, STAT1 and STAT3 through induction of the cyclin D1 and MAPK/ERK kinase pathways. High-throughput proteomic analysis led us to identify AKT as a central hub of the Trop-2 activation network in human cancer cells. AKT inhibitors only blocked the growth of Trop-2–expressing tumors, but were ineffective on Trop-2–null cells, indicating Trop-2 as a pivotal AKT activator for tumor growth (48). AKT was also shown to be central to Trop-2 signaling in lung adenocarcinomas, as inhibition of *TROP2* expression, *via* DNA methylation, enacted IGF-1R signaling and AKT/β-catenin (43). These signaling pathways were shown to be triggered by a Trop-2, Na+/K+ ATPase, CD9, PKCα, cofilin membrane signaling super-complex. This super-complex was found to be ubiquitous, but essentially dormant in normal cells, and was shown to be activated by Trop-2 to trigger colorectal cancer growth and invasion (29).

These findings suggested functional interaction and potential synergy of mutagenic and non-mutagenic cancer drivers. Trop-2-triggered PKCα, tetraspanins, AKT, Jun, NF-κB, Rb, STAT1, STAT3, cyclin D1, MAPK/ERK kinases fall within main hallmarks of tumor progression (6, 18, 49), and comprise signaling networks triggered by mutated oncogenes (1). As indicated above, DNA methylation and *TP53* status are instrumental to *TROP* gene copy number variation and to consequent aberrant overexpression of wtTrops (21, 41).

Consistent with this, genomic loss of *mTrop2* rendered *Arf*-null mice susceptible to the formation of biphasic sarcomatoid carcinomas upon carcinogen exposure in skin cancer-resistant mouse strains (C57BL/6). Ras-transformed keratinocytes derived from *mTrop2*(-/-)*Arf*(-/-) mice exhibit enhanced proliferative and migratory capacity as well as increased activation of MAPK and Src (19). Ras-transformed *mTrop2*(-/-) keratinocytes were shown to undergo epithelial to mesenchymal transition (EMT) and formed tumors with spindle cell histology. Consistent with this, *TROP2* mRNA levels were found to be down-regulated in head and neck squamous cell carcinomas undergoing EMT (19). Mutated Ras was shown to synergize with the *TROP2-CYCLIND1* mRNA chimera in transforming primary cells *in vitro* and in inducing tumor growth *in vivo* (20). The vast overexpression of Trop-2 in main cancer types, e.g. breast (76%), stomach (70%), prostate (80%), pancreas (83%), cervix (91%) cancers (23), indicates that these mutagenic and non-mutagenic signatures are candidate to frequently interact in cancer cells (1).

### 2.6 Mutagenic and Trop-2 non-mutagenic signatures – the pancreatic cancer case

**Figure 3:**
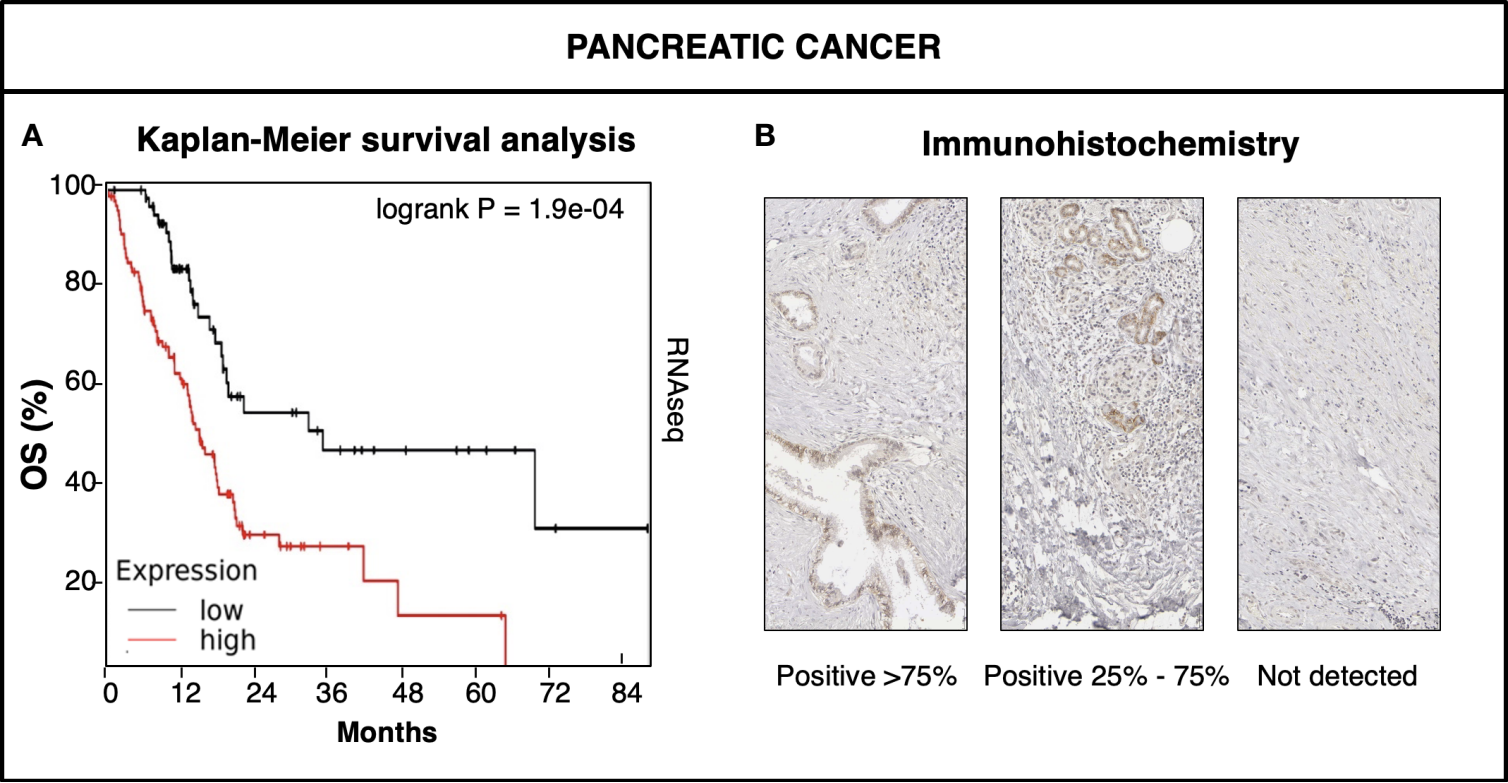
Trop-2 impact on pancreatic cancer. **(A)** KMPlot database (www.kmplot.com) data (50) of expression of *TROP2* mRNA in pancreatic cancer versus patient overall survival (OS). RNAseq high (red) versus low (black) *TROP2* mRNA levels were compared by Kaplan-Meier survival analysis. Logrank P value of survival curve comparison is shown. **(B)** Immunohistochemical analysis of Trop-2 protein expression in representative cases of pancreatic cancer. Percent expression of Trop-2 by cancer cells is indicated. Expression classes were categorized as high (>75% of cells) medium (25-75% of cells) and low/nil (<25% of cells) (23).

A prognostic impact of Trop-2 and of activation-inducing ADAM10 was found in major clinically-relevant cancer types (8, 11, 16). Among them, Trop-2 was shown to impact on the progression and metastatic relapse of pancreatic cancer (PC) (**Figure 3**) (10, 11). PC is a very aggressive disease with a poor prognosis (5-year survival: ∼ 6%). Recent evidence on mutagenic and non-mutagenic signatures in PC has started shedding light on its pathogenic determinants. *KRAS* mutations have been detected in about 90% of pre-neoplastic non-invasive lesions (low-grade pancreatic intraepithelial neoplasia) (51), though more rarely at more advanced stages of PC, suggesting a ‘common ground’ for early PC development. Data on *TROP2* prevalence added to this scenario, as overexpression of Trop-2 was found in the majority of PC patients (10). *TROP2* mRNA and protein levels were subsequently shown to be sharp prognostic biomarkers for PC (16) (**Figure 3**). These findings suggested a driving role of Trop-2 in PC (16) and a convergent impact of *TROP2* and *KRAS* signaling pathways (17) at early stages of cancer development (23). Along PC grade progression, additional mutations were shown to occur in cell growth/death regulatory genes including *CDKN2A* and *TP53*. Invasive PC carry mutations in *SMAD4* in about 55% of the cases (51), suggesting again a broad, common ground for PC development.

Aberrant DNA methylation of gene promoter CpG islands was detected in *CACNA1G, CDH1, CDKN2A, DAPK1, MGMT, MINT1-2-31-32, MLH1, RARB, THBS1* and *TIMP3* (52, 53). Hypomethylation of the *CLDN4, LCN2, MSLN, PSCA, S100A4, TFF2* and *YWHAS* genes, with corresponding high levels of transcription in PC, was found in parallel (54–56). Consistent with findings that methylation of *BRCA1* is associated to metastatic relapse (57), promoter methylation of *BRCA1* was found in 46% of PC (58), suggesting a driving role in PC occurrence and progression (59, 60). Parallel impact was shown for *BRCA-1* and *BRCA-2* mutations (59, 60), suggesting a convergent mechanism of inactivation of shared target genes in PC. DNA methylation interacts with modified histones and plays a role in chromatin remodeling and heterochromatin formation (61). Unsurprisingly, mutations of the *SWI/SNF* chromatin remodeling genes were found in 15% of PC cases (51), suggesting impact on downstream epigenetic changes.

These findings suggested a mechanistic overlap between the Trop-2 signature and mutagenic/epigenetic PC driving changes, and supports a model of PC progression, through accumulation of genetic and epigenetic changes during tumor initiation, promotion and progression (62). Corresponding impact of genetic and epigenetic changes was demonstrated for PC response to therapy (63, 64), extending the value of Trop-2 as a therapy target in PC (33), through the targeting of the Trop-2 activated form by next-generation monoclonal antibodies (34).

## 3 Conclusions

Modelling of cancer genetic signatures, according to a multi-stage, somatic mutation-based carcinogenesis process, has generated key insight on cancer development, and has led to major advances of anticancer therapy (6, 65). However, mutation-only tumor progression models may risk missing major regulatory paths and networks, that are driven by epigenetic components (6), together with non-mutagenic (23), cancer-prone, acquired phenotypes (16, 66).

Upregulation of Trop-2 was shown to quantitatively stimulate human cancer growth (23) and metastasis (16) in the absence of detectable *TROP2* gene mutations. Acquisition of stable, potentially heritable modes of gene expression, as driven by gene expression regulatory loops/miRNA (45, 46) and by post-transcriptional/post-translational events, like oncogenic mRNA chimeras (20, 67, 68) were shown to contribute to key steps of oncogenic transformation. Taken together, these findings suggest that mutagenic and the *TROP2* non-mutagenic pathways deeply intertwine in driving cancer cell growth, for a pivotal role in tumor progression in solid cancers. Next-generation identification of oncogenic mutagenic/non-mutagenic signatures in cancer development may provide additional insight and generate novel models of cancer development.

## Data availability statement

The datasets presented in this study can be found in online repositories. The names of the repository/repositories and accession number(s) can be found in the article/supplementary material.

## Author contributions

SA designed the strategy of the article. EG, RDP, GS, SA contributed to data collection, writing of the article and revision of the final text. All authors contributed to the article and approved the submitted version.
